# Surgery-first approach reduces the overall treatment time without damaging long-term stability in the skeletal class III correction: a preliminary study

**DOI:** 10.1186/s40902-021-00304-8

**Published:** 2021-07-17

**Authors:** Young-Wook Park, Kwang-Jun Kwon, Yei-Jin Kang, In-San Jang

**Affiliations:** 1 Department of Oral and Maxillofacial Surgery, College of Dentistry, Gangneung-Wonju National University, 7, Jukheon-gil, Gangneung-si, Gangwon-do 25457 South Korea; 2 Department of Orthodontics, College of Dentistry, Gangneung-Wonju National University, Gangneung, South Korea

**Keywords:** Skeletal class III correction, Surgery-first approach, Long-term skeletal stability, Overall treatment time

## Abstract

**Background:**

Compared to the conventional approach, including preoperative orthodontic preparation, the so-called surgery-first approach (SFA) seems to reduce the overall treatment time in the correction of skeletal class III dentofacial deformity. However, there have been controversies about postoperative skeletal stability with SFA. Therefore, we investigated the long-term stability and the overall treatment time after maxillomandibular surgery for skeletal class III correction with or without preoperative orthodontic preparation.

**Methods:**

This retrospective study included eight patients who underwent maxillomandibular surgery for class III correction with the SFA (SFA group) and 20 patients who underwent the conventional approach (CA group). A comparative study of the change in the maxillary and mandibular position on preoperative (T1), 1-day (T2), 6-month (T3), and 2-year (T4) postoperative lateral cephalograms. We calculated the overall treatment time for each group.

**Results:**

At the presurgical stage (T1), there was no bias in the skeletal features between the two groups. In the surgical change from T1 to T2, the mandible (point B) of the CA group was significantly moved superiorly. Short-term changes from T2 to T3 revealed that the mandible moved forward in both groups, whereas the maxillary position showed no significant changes. Long-term changes from T3 to T4 demonstrated that none of the measured parameters showed any significant differences. Finally, the average of overall treatment time was 15.1 months in the SFA group and 26.0 months in the CA group.

**Conclusions:**

These findings suggest that SFA in bimaxillary orthognathic surgery for skeletal class III correction leads to predictable long-term skeletal stability, similar to surgery with CA. Furthermore, SFA reduced the overall treatment time compared to CA.

## Background

Conventionally, orthognathic surgery consists of three phases: preoperative orthodontic treatment, operation, and postoperative orthodontic treatment. However, oral and maxillofacial surgeons have tried to reverse the conventional phases of orthodontic treatment after the surgical change of the skeletal base to consider patients’ social and psychological demands. Through clinical trials, “the surgery-first approach” (SFA), which involves surgery plus postoperative orthodontic treatment without preoperative orthodontic preparation, has been proposed [1].

With an SFA, patients can acquire the esthetically improved face without lengthy preoperative orthodontic treatment. In addition, preceding bone surgery triggers an intense osteoclastic activity and sequential metabolic alterations, including increased bone turnover and decreased mineral density, which accelerates the decompensation process of postoperative orthodontic treatment [2]. Additionally, the surrounding soft tissues such as the tongue, lips, and masticatory muscles seem to facilitate postoperative tooth movement [3]. Consequently, this surgery-facilitated orthodontics can reduce the overall duration of surgico-orthodontic treatment [4]. However, the anticipated reduction in overall treatment time may be related to a more efficient protocol of SFA.

Another issue is postoperative skeletal stability as well as the inclusion criteria for patients with SFA. To the best of our knowledge, the short-term and long-term skeletal stability of SFA is still debatable and questionable [5–8]. Therefore, some clinicians have proposed restricted and controlled indications for the application of SFA [9]. However, the SFA is constantly evolving with extended indications [10, 11] to aid new concepts and treatment technologies. Therefore, this study aimed to evaluate the long-term skeletal stability and overall treatment time after bimaxillary surgery for skeletal class III correction with SFA.

## Methods

This retrospective study was approved by the Institutional Review Board of Gangneung-Wonju National University Dental Hospital (GWNUDH-IRB2019-A009). Orthognathic surgeries were performed by a single surgeon between 2009 and 2013. The inclusion criteria for patients were as follows:

1. The patient was diagnosed with developmental dentofacial deformity, without any congenital anomalies or traumatic deformity.
2. Patients with class III deformity with or without facial asymmetry.
3. Patients who underwent maxillomandibular surgery using standard Le Fort I maxillary osteotomy and bilateral mandibular SSRO.
4. Osteofixation was achieved using absorbable poly l-lactic acid (PLLA; BioSorb FX, Bionix Implants Inc., Finland) plates and screws.
5. Patients with a series of lateral cephalograms for at least 2 years after orthognathic surgery.

All patients underwent surgical alterations of the maxilla with unilateral impaction and/or posterior impaction for clockwise rotation of the maxillomandibular segment. For maxillary fixation in both groups, four 2.0 mm, 7-holed L-type plates were used on the standard position. Patients were assigned into two groups according to the presence of preoperative orthodontic treatment: the SFA group (SFA, *n* = 8) and the conventional approach group (CA, *n* = 20). In the SFA group, the bonding procedure for intermaxillary fixation was performed immediately before the orthognathic surgery, and 25 units of botulinum toxin (Meditoxin®, Medy-Tox, Korea) into the masseter muscles bilaterally to decrease the muscular force.

A comparative study of the change in the maxillary and mandibular position was performed on preoperative (T1), 1-day (T2), 6-months (T3), and 2-years (T4) postoperative lateral cephalograms using a photo-analysis software, Xelis dental® (Infinity care, Seoul, Korea).

**Fig. 1:**
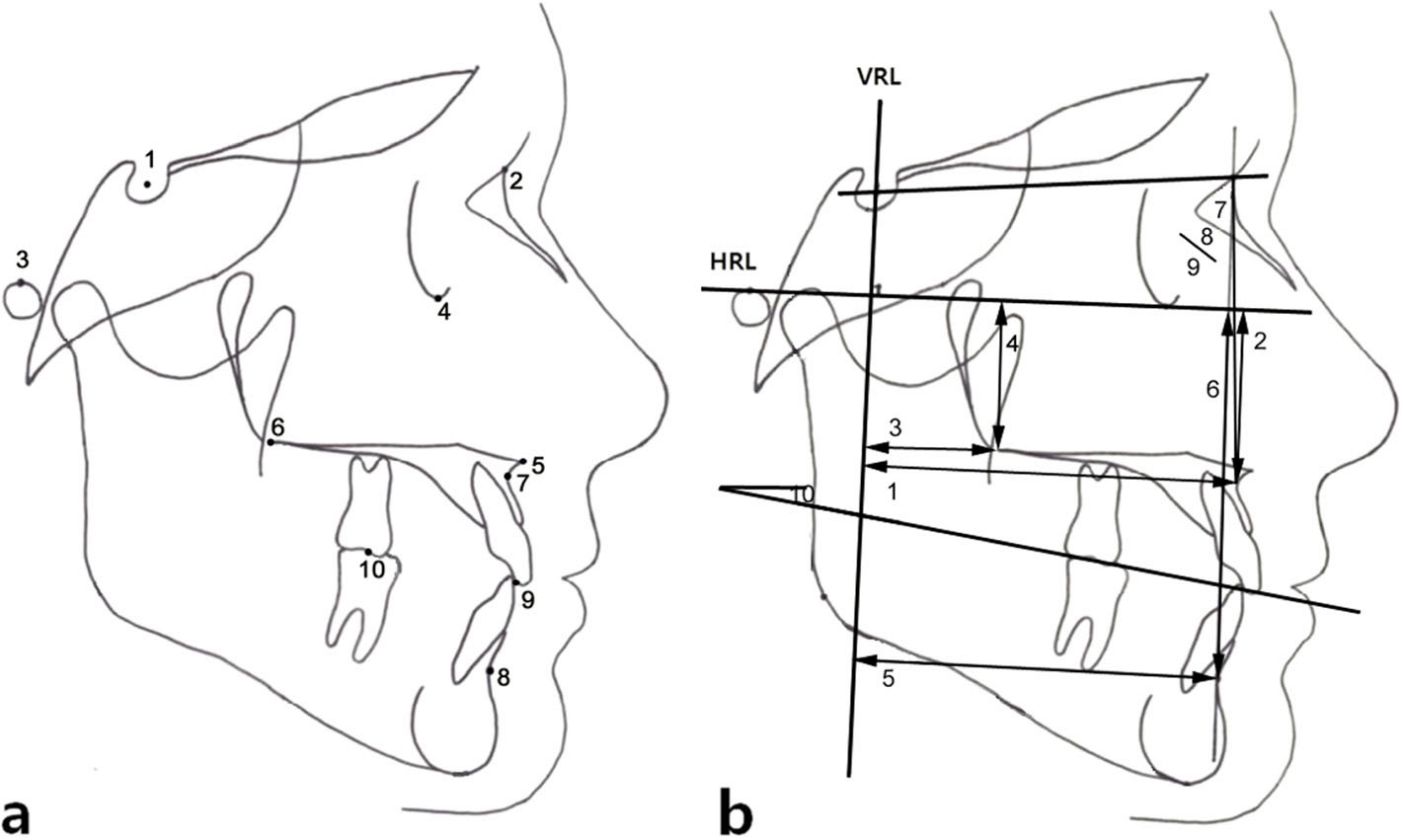
**a** Reference points used in this study: 1. Sella (S); 2. Nasion (N); 3. Porion (Po); 4. Orbitale (Or); 5. Anterior nasal spine (ANS); 6. Posterior nasal spine (PNS); 7. Subspinale (A); 8. Supramentale (B); 9. The point bisects the vertical distance between the tips of maxillary (U1) and mandibular central incisor (L1) 10. The point bisects the vertical distance between the occlusal surfaces of maxillary (U6) and mandibular first molar (L6). **b** Measuring parameters used in this study: 1. VRL-A (mm); 2. HRL-A (mm); 3. VRL-PNS (mm); 4. HRL-PNS (mm); 5. VRL-B (mm); 6. HRL-B (mm); 7. SNA (°); 8. SNB (°); 9. ANB (°); 10. Occlusal plane angle (°)

We set up ten reference points (Fig. 1a) and ten measuring parameters (Fig. 1b) in consecutive lateral cephalograms. We used the FH plane (porion; Po-orbitale; Or) as the horizontal reference line, and the vertical reference line was defined as the line perpendicular to the FH plane over the Sella (S) point. Regarding the occlusal plane, we used the point bisecting the vertical distance between the tips of the maxillary and mandibular central incisors and between the occlusal surfaces of the maxillary and mandibular first molars [12].

Statistical analyses were performed using IBM SPSS Statistics version 23 (IBM Co., NY, USA). The differences between T1 and T2 (T2-T1) determined the surgical change, the differences between T2 and T3 (T3-T2) determined the short-term relapse, and the differences between T3 and T4 (T4-T3) determined the long-term relapse after surgery. The Wilcoxon rank-sum test was used to analyze the surgical changes and short-term and long-term relapses. The Mann-Whitney test was used to analyze differences between groups at each time point. Statistical significance was set at *p* < 0.05.

In addition, we analyzed the total treatment time for each group. In the SFA group, we calculated the treatment duration from the day of surgery to the day of debonding. In the CA group, we calculated the treatment duration from the day of bonding to the day of debonding, and a comparison of the mean value was performed using an independent t test.

## Results

A total of 28 patients (15 males and 13 females) were enrolled in this retrospective study. The patients’ basic information is summarized in Table 1. Clinically, no operative complications were observed in any patient.

**Table 1:** Patient’s information

	SFA group (*n* = 8)	CA group (*n* = 20)
**Age** (Years)	19.4 ± 1.41	22.2 ± 3.17
**Gender**		
Male	3	12
Female	5	8

### Preoperative stage

In the preoperative stage, no statistical differences were observed in any of the parameters between the two groups (Table 2). Therefore, the skeletal characteristics of patients did not differ between the two groups.

**Table 2:** Comparison of Experimental group and Control group on the pre-surgical stage (T1)

Parameters	SFA group (*n* = 8)		CA group (*n* = 20)		*P* value
	Mean	SD	Mean	SD	
**Horizontal measurements**					
VRP-A (mm)	58.61	3.32	59.73	5.40	0.328
VRP-B (mm)	60.49	5.90	62.59	7.08	0.601
VRP-PNS (mm)	15.76	3.17	16.50	3.05	0.566
**Vertical measurements**					
HRP-A (mm)	33.06	3.72	33.35	4.86	1.000
HRP-B (mm)	80.37	3.84	78.35	7.34	0.409
HRP-PNS (mm)	23.74	2.15	23.53	3.69	0.980
**Angular measurements**					
SNA (°)	83.48	4.80	81.63	3.54	0.304
SNB (°)	86.07	6.86	84.60	3.95	0.940
ANB (°)	−2.59	3.07	−2.96	2.60	0.533
Occlusal plane angle (°)	12.50	4.00	10.87	3.55	0.636

*P* = 0.050

Mann-Whitney U test

### Surgical change (T2-T1)

After the operation, point B was significantly moved posteriorly according to the surgical change of the mandibular setback in both groups. However, point B was significantly moved superiorly in the CA group. SNB was significantly decreased, and ANB was significantly increased in both groups. Otherwise, the PNS significantly moved superiorly in the SFA group. The occlusal plane angle significantly increased only in the CA group (Table 3).

**Table 3:** Comparison of surgical change (T2-T1) on Experimental group and Control group

Parameters	SFA group (*n* = 8)		CA group (*n* = 20)		*P* value
	Mean	*P* value	Mean	*P* value	
**Horizontal measurements**					
VRP-A (mm)	0.87 ± 2.46	0.575	0.63 ± 2.98	0.360	0.980
VRP-B (mm)	−9.30 ± 4.88	0.012^a^	−9.51 ± 6.46	0.000^a^	0.709
VRP-PNS (mm)	0.69 ± 3.48	0.674	−0.36 ± 3.55	0.723	0.636
**Vertical measurements**					
HRP-A (mm)	−0.49 ± 4.87	0.484	0.50 ± 3.81	0.809	0.533
HRP-B (mm)	−1.66 ± 2.26	0.080	−2.41 ± 3.23	0.003^a^	0.672
HRP-PNS (mm)	−3.04 ± 2.35	0.017^a^	−1.77 ± 4.33	0.093	0.438
**Angular measurements**					
SNA (°)	0.14 ± 4.88	0.263	1.25 ± 2.56	0.052	0.746
SNB (°)	−5.88 ± 3.58	0.012^a^	−4.79 ± 3.05	0.000^a^	0.469
ANB (°)	6.02 ± 3.05	0.012^a^	6.04 ± 2.24	0.000^a^	0.709
Occlusal plane angle (°)	2.01 ± 3.66	0.208	3.61 ± 4.72	0.004^a^	0.636

*P* = 0.05

Wilcoxon rank sum test

Mann-Whitney U test

^a^Significant difference

### Short-term change (T3-T2)

At 6 months postoperatively, point B moved to a horizontally anterior position only in the SFA group. In the CA group, point A was significantly moved superiorly, SNB increased, and ANB decreased (Table 4).

**Table 4:** Comparison of post-surgical change (T3-T2) on Experimental group and Control group

Parameters	SFA group (*n* = 8)		CA group (*n* = 20)		*P* value
	Mean	*P* value	Mean	*P* value	
**Horizontal measurements**					
VRP-A (mm)	−0.19 ± 1.84	1.000	−0.43 ± 3.48	0.867	0.980
VRP-B (mm)	1.96 ± 2.09	0.036^a^	1.03 ± 2.64	0.411	0.980
VRP-PNS (mm)	0.25 ± 1.78	1.000	0.14 ± 1.53	0.614	0.901
**Vertical measurements**					
HRP-A (mm)	−0.72 ± 2.03	0.575	−2.78 ± 2.69	0.001^a^	0.110
HRP-B (mm)	−2.21 ± 4.75	0.208	0.37 ± 2.89	0.737	0.218
HRP-PNS (mm)	−0.25 ± 1.63	0.735	−0.86 ± 4.47	0.837	0.784
**Angular measurements**					
SNA (°)	0.54 ± 2.96	0.674	0.27 ± 1.02	0.287	0.258
SNB (°)	1.81 ± 2.63	0.069	1.30 ± 1.24	0.002^a^	0.746
ANB (°)	−1.27 ± 1.55	0.123	−1.02 ± 1.44	0.005^a^	0.438
Occlusal plane angle (°)	0.60 ± 2.14	0.208	−1.48 ± 4.93	0.313	0.328

*P* = 0.05

Wilcoxon rank sum test

Mann-Whitney U test

^a^Significant difference

### Long-term change (T4-T3)

At 2 years postoperatively, none of the measured parameters showed any significant differences compared to 6 months postoperatively. In addition, none of the measured parameters showed any significant differences between the two groups (Table 5).

**Table 5:** Comparison of long-term result change (T4-T3) on Experimental group and Control group

Parameters	SFA group (*n* = 8)		CA group (*n* = 20)		*P* value
	Mean	*P* value	Mean	*P* value	
**Horizontal measurements**					
VRP-A (mm)	0.97 ± 1.37	0.173	0.16 ± 1.81	0.831	0.286
VRP-B (mm)	1.79 ± 2.96	0.249	1.23 ± 3.53	0.193	0.708
VRP-PNS (mm)	−0.38 ± 0.77	0.249	0.42 ± 1.31	0.381	0.177
**Vertical measurements**					
HRP-A (mm)	−0.97 ± 1.47	0.173	−0.03 ± 1.57	1.000	0.256
HRP-B (mm)	−0.18 ± 2.96	0.917	−0.07 ± 1.35	0.981	0.812
HRP-PNS (mm)	−0.22 ± 1.21	0.917	0.05 ± 1.22	0.981	1.000
**Angular measurements**					
SNA (°)	−0.44 ± 2.77	0.917	0.24 ± 0.91	0.227	0.392
SNB (°)	−0.23 ± 2.70	0.463	0.34 ± 1.41	0.356	1.000
ANB (°)	−0.21 ± 1.05	0.753	−0.10 ± 0.75	0.670	0.865
Occlusal plane angle (°)	−2.22 ± 1.77	0.075	−0.24 ± 2.00	0.523	0.087

*P* = 0.05

Wilcoxon rank sum test

Mann-Whitney U test

### Total treatment time

The average of total treatment time in the CA group was 26.0 ± 9.5 months. On the contrary, the average of total treatment time in the SFA group was 15.1 ± 2.4 months. Therefore, the total treatment time was significantly lower in the SFA group than in the CA group (*p* < 0.05).

## Discussion

This study was performed to determine the long-term maxillomandibular positional stability after bimaxillary surgery with SFA to correct the skeletal class III deformity, which required a clockwise rotation of the maxillomandibular complex. One of the most important purposes of orthognathic surgery is to improve facial balance and esthetics. By SFA, orthognathic patients can improve their facial balance from the beginning of the surgico-orthodontic treatment and take advantage of shortening the total treatment time. In this study, all patients in the SFA group were satisfied with their early changes in facial esthetics and shortened treatment duration.

Originally, SFA was proposed for only mild to moderate skeletal class III deformities. However, the scope of SFA has recently expanded to bimaxillary surgery [13], including treatment of asymmetrical skeletal class III deformities [11]. In our study, the number of patients with apparent facial asymmetry showing a difference of 3 mm or more in the amount of mandibular setback on each side was four in the SFA group and nine in the CA group. We excluded patients who showed severe anterior open bite or severe mandibular asymmetry in the SFA group. However, statistically, there was no bias in the facial features between the SFA and CA groups.

All operations were bimaxillary procedures accompanied by a posterior impaction or unilateral impaction of the maxillary segment. Therefore, we considered a forward movement of the mandible and inferior movement of the maxilla as the direction of relapse. The amount of mean setback movement of the mandible was 9.3 mm in the SFA group and 9.5 mm in the CA group. In addition, the amount of mean superior movement of the PNS was 3.0 mm in the SFA group and 1.7 mm in the CA group, which had no significant difference statistically. Many patients in both groups underwent genioplasty, which did not affect the position of point B in our surgical design. Osteofixation was achieved by absorbable PLLA, which demonstrated predictable skeletal stability compared to that of titanium plating systems [14]. Immediately after the orthognathic procedure, we injected routine dosage of botulinum toxin into the masseter muscles bilaterally to decrease the muscular force [15].

In the surgical change, point B was significantly moved superiorly in the CA group but not in the SFA group, although maxillary impaction had been performed. This is because the vertical dimension of occlusion in the SFA group, which means the superior-inferior relationship of the maxilla and the mandible when the teeth are occluded in surgical stent, was increased to avoid occlusal interference due to unstable surgical occlusion. For the same reason, the occlusal plane angle significantly increased only in the CA group. Other surgical changes in this study were based on the maxillomandibular segmental movements from the surgical plan of grossly maxillomandibular clockwise rotation.

As the increased vertical dimension of occlusion in the SFA group decreased during the period of postoperative orthodontic treatment, the mandible was moved anteriorly as a result of the counterclockwise rotation. Therefore, at 6 months postoperatively, point B was moved to a horizontally anterior position only in the SFA group, indicating a short-term relapse. Actual horizontal changes of point B were 1.96 ± 2.09 mm in the SFA group and 1.03 ± 2.64 mm in the CA group. In contrast, in the CA group, SNB was increased, and ANB was decreased, which also suggests that point B was moved anteriorly. Actual changes of SNB were 1.81 ± 2.63° in the SFA group and 1.30 ± 1.24° in the CA group, which can be considered in the normal range of relapse in BSSRO and mandibular setback surgery [16]. However, most parameters related to the maxillary change had no significance in either group, except that it was significantly moved superiorly only in the CA group. The CA group had relatively stable occlusion with almost full-size orthodontic archwires after surgery. Therefore, the application of class III elastics during postoperative orthodontic treatment might be more easily transferred to the maxilla, resulting in the displacement of point A.

Mah et al. reported greater horizontal and vertical relapse at the time point of postoperative 1 year or debonding in the surgery-first orthognathic approach for skeletal class III correction [17]. They suggested that the postoperative counterclockwise rotation of the mandible in the surgery-first orthognathic approach causes relapse. However, Park et al. reported no significant differences in terms of postoperative stability at 6 months after bimaxillary surgery for skeletal class III malocclusion, either with or without preoperative orthodontic treatment [18]. They found that the similar relapse rates between both groups were due to their standardized rigid inclusion criteria for surgery-first bimaxillary surgery.

In another clinical study, Han et al. reported additional horizontal relapse of the mandible until the time point of debonding (16.6 months after surgery) after mandibular setback surgery in an SFA [19]. Therefore, they suggested that it is necessary to consider mandibular forward movement from the increase in the vertical dimension of surgical occlusion and additional relapse in the SFA. However, in this study, all measuring parameters related to long-term relapse did not show any significant change in the SFA group at the time point of postoperative 2 years. In our practice, the average debonding time was 14.7 months after surgery in the SFA group. Therefore, we believe that the unstable surgical occlusion in the SFA group stabilized within 6 months postoperatively. Taken together, after 6 months postoperatively, there was no additional skeletal relapse in our surgery-first protocol.

The key is how the temporary instability of the surgically created occlusion is resolved in postsurgical treatment. We successfully overcame unstable surgical occlusion in the SFA group by stable osteofixation at the osteotomy site and application of our protocol during orthodontic treatment. At initial diagnosis, we applied the SFA using 3D-CBCT (Alphard VEGA, ASAHI ROENTGEN, Japan) virtual imaging and simulation surgery to predict accurate postsurgical tooth alignment [20]. To cover the unstable surgical occlusion, we initiated orthodontic movements after 1 month of stabilization. Therefore, we can use the active period of surgery-induced, regionally accelerated phenomenon, which is known to be active 3–4 months after surgery [21]. We maintained a consistent skeletal relationship using effective interocclusal elastics. Lastly, the decreased masseter muscle thickness from the injection of the botulinum toxin [22] seems to mitigate the undesirable force from the masseter muscle, which acts in the relapse tendency.

One of the advantages of SFA is that it shortens the duration of surgico-orthodontic treatment. This study demonstrated that the average of total treatment time in the CA group was 26.0 months (range, 12‑42 months). In contrast, the average of total treatment time in the SFA group was 15.1 months (range, 9‑24 months). From a clinical point of view, extraction of the maxillary premolars and closing the extraction sites for orthodontic purposes might be the most influential factor on the total treatment time. In this study, the number of orthodontic cases with premolar extraction was two out of eight in the SFA group and 14 out of 20 in the CA group. In conventional treatment planning, severe crowding of the anterior teeth is ruled out for SFA. Therefore, the percentage of tooth extraction was higher in the CA group than in the SFA group, which affected the total treatment time. A prospective study regarding the treatment period for skeletal class III dentofacial deformities reported that the average of total treatment period of the SFA was shorter (14.6 months) than the CA (22.0 months). Thus, SFA dramatically reduced treatment time [23]. Other studies have suggested that the observed reduction in total treatment duration is related to more efficient orthodontic protocols, such as skeletal anchorage or palatal arch expansion [24, 25].

In summary, this study demonstrated the tendency of short-term relapse in the mandibular position in the correction of skeletal III deformity regardless of preoperative orthodontic preparation. In particular, the actual change in point B was greater in the SFA group than in the CA group due to unstable surgical occlusion. However, this temporary instability was easily resolved within 6 months after the operation, without damaging long-term mandibular stability. In addition, the maxillary position remained stable throughout the course of surgico-orthodontic treatment. However, the limitation of this study is the reliability of the statistics due to the small sample size of the SFA group. And the data was analyzed by 2-dimensional measurements only, not the 3-dimensional ones. Therefore, a prospectively designed study with larger sample size is needed. Intimate cooperation between the orthognathic surgeon and the orthodontist is crucial to achieving a successful result in SFA due to the absence of preoperative occlusal preparation.

## Conclusion

A greater amount of short-term relapse due to counterclockwise rotation of the mandible was observed in the SFA group. However, no additional long-term relapses were observed. Therefore, we can preliminarily conclude that SFA in bimaxillary orthognathic surgery for skeletal class III correction leads to predictable long-term skeletal stability, comparable to the CA. Additionally, patients are satisfied with the early improvement of facial esthetics and shortened treatment time. Therefore, SFA can be a useful and promising treatment paradigm for the management of skeletal class III deformities.

## Data Availability

All datasets used in this study were shown in this paper.

## Abbreviation

n: Number

## References

[1] Hong KJ, Lee JG (1999). 2 Phase treatment without preoperative orthodontics in skeletal class III malocclusion. Korean J Oral Maxillofac Surg.

[2] Liou EJ, Chen PH, Wang YC (2011). Surgery-first accelerated orthognathic surgery: postoperative rapid orthodontic tooth movement. J Oral Maxillofac Surg.

[3] Behrman SJ, Behrman DA (1988). Oral surgeon’s considerations in surgical orthodontic treatment. Dent Clin North Am.

[4] Hernandez-Alfaro F, Guijarro-Martinez R, Molina-Coral A, Badia-Escriche C (2011). “Surgery first” in bimaxillary orthognathic surgery. J Oral Maxillofac Surg.

[5] Ko EW, Lin SC, Chen YR, Huang CS (2013). Skeletal and dental variables related to the stability of orthognathic surgery in skeletal class III malocclusion with a surgery-first approach. J Oral Maxillofac Surg.

[6] Yang L, Xiao YD, Liang YJ (2017). Does the surgery-first approach produce better outcomes in orthognathic surgery? A systematic review and meta-analysis. J Oral Maxillofac Surg.

[7] Wei H, Liu Z, Zang J, Wang X (2018). Surgery-first/early-orthognathic approach may yield poorer postoperative stability than conventional orthodontics-first approach: a systematic review and meta-analysis. Oral Surg Oral Med Oral Pathol Oral Radiol.

[8] Jeong WS, Lee JY, Choi JW (2018). Large-scale study of long-term vertical skeletal stability in a surgery-first orthognathic approach without presurgical orthodontic treatment: part II. J Craniofac Surg.

[9] Liou EJ, Chen PH, Wang YC (2011). Surgery-first accelerated orthognathic surgery: orthodontic guidelines and setup for model surgery. J Oral Maxillofac Surg.

[10] Liao YF, Chiu YT, Huang CS, Ko EW, Chen YR (2010). Presurgical orthodontics versus no presurgical orthodontics: treatment of outcome of surgical-orthodontic correction for skeletal class III open bite. Plast Reconstr Surg.

[11] Liao YF, Chen YF, Yao CF, Chen YA, , Chen YR (2018) Long-term outcomes of bimaxillary surgery for treatment of asymmetric skeletal class III deformity using surgery-first approach. Clin Oral Invest. 10.1007/s00784-018-2603-y 30155572

[12] Li JL, Chung HK, Wang M (2014). Changes of occlusal plane inclination after orthodontic treatment in different dentoskeletal frames. Progress Orthodon.

[13] Park HM, Lee YK, Choi JY, Baek SH (2014). Maxillary incisor inclination of skeletal class III patients treated with extraction of the upper first premolars and two-jaw surgery: conventional orthognathic surgery vs surgery-first approach. Angle Orthod.

[14] Park JM, Park YW (2010). Postoperative stability of fixation with absorbables in simultaneous maxillomandibular orthognathic surgery. Maxillofac Plast Reconstr Surg.

[15] Kang YJ, Cha BK, Choi DS, Jang IS, Kim SG (2019). Botulinum toxin-A injection into the anterior belly of the digastric muscle for the prevention of post-operative open bite in class II malocclusions: a case report and literature review. Maxillofac Plast Reconstr Surg.

[16] Costa F, Robiony M, Politi M (2001). Stability of sagittal split ramus osteotomy used to correct class III malocclusion: review of the literature. Int J Adult Orthodon Orthognath Surg.

[17] Mah DH, Kim SG, Oh JS (2017). Comparative study of postoperative stability between conventional orthognathic surgery and a surgery-first orthognathic approach after bilateral sagittal split ramus osteotomy for skeletal class III correction. J Korean Assoc Oral Maxillofac Surg.

[18] Park KH, Sandor GK, Kim YD (2016). Skeletal stability of surgery-first bimaxillary orthognathic surgery for skeletal class III malocclusion, using standardized criteria. Int J Oral Maxillofac Surg.

[19] Han JJ, Lin SC, Jung S (2019). Evaluation of postoperative mandibular positional changes after mandibular setback surgery in a surgery-first approach: isolated mandibular surgery versus bimaxillary surgery. J Oral Maxillofac Surg.

[20] Choi DS, Garagiola U, Kim SG (2019). Current status of the surgery-first approach (part I): concept and orthodontic protocols. Maxillofac Plast Reconstr Surg.

[21] Zingler S, Hakim E, Finke D (2017). Surgery-first approach in orthognathic surgery: psychological and biological aspects - a prospective cohort study. J Craniomaxillofac Surg.

[22] Moon YM, Kim YJ, Kim MK (2015). Early effect of Botox-A injection into the masseter muscle of rats: functional and histological evaluation. Maxillofac Plast Reconstr Surg.

[23] Jeong WS, Choi JW, Kim DY, Lee JY, Kwon SM (2017). Can a surgery-first orthognathic approach reduce the total treatment time?. Int J Oral Maxillofac Surg.

[24] Baek S, Ahn HW, Kwon YH, Choi JY (2010). Surgery-first approach in skeletal class III malocclusion treated with 2-jaw surgery: evaluation of surgical movement and postoperative orthodontic treatment. J Craniofac Surg.

[25] Villegas C, Uribe F, Sugawara J, Nanda R (2010). Expedited correction of significant dentofacial asymmetry using a “surgery first” approach. J Clin Orthod.

